# Population Genetic Structure of *Listeria monocytogenes* Strains Isolated From the Pig and Pork Production Chain in France

**DOI:** 10.3389/fmicb.2018.00684

**Published:** 2018-04-06

**Authors:** Benjamin Félix, Carole Feurer, Aurelien Maillet, Laurent Guillier, Evelyne Boscher, Annaëlle Kerouanton, Martine Denis, Sophie Roussel

**Affiliations:** ^1^Maisons-Alfort Laboratory for Food Safety, Salmonella and Listeria Unit, University of Paris-Est, French Agency for Food, Environmental and Occupational Health & Safety (ANSES), Maisons-Alfort, France; ^2^The French Institute for Pig and Pork Industry, IFIP, Le Rheu, France; ^3^Hygiene and Quality of Poultry and Pig Products Unit, Bretagne Loire University, French Agency for Food, Environmental and Occupational Health & Safety (ANSES), Ploufragan, France

**Keywords:** *Listeria monocytogenes*, pig, pork, PFGE, MLST, population structure, genetic diversity

## Abstract

*Listeria monocytogenes* is an ubiquitous pathogenic bacterium, transmissible to humans through the consumption of contaminated food. The pork production sector has been hit hard by a series of *L. monocytogenes*-related food poisoning outbreaks in France. An overview of the diversity of strains circulating at all levels of the pork production chain, from pig farming (PF) to finished food products (FFP), is needed to identify the contamination routes and improve food safety. Until now, no typing data has been available on strains isolated across the entire pig and pork production chain. Here, we analyzed the population genetic structure of 687 *L. monocytogenes* strains isolated over the last 20 years in virtually all the French *départements* from three compartments of this production sector: PF, the food processing environment (FPE), and FFP. The genetic structure was described based on Multilocus sequence typing (MLST) clonal complexes (CCs). The CCs were obtained by mapping the PFGE profiles of the strains. The distribution of CCs was compared firstly between the three compartments and then with CCs obtained from 1106 strains isolated from other food production sectors in France. The predominant CCs of pig and pork strains were not equally distributed among the three compartments: the CC37, CC59, and CC77 strains, rarely found in FPE and FFP, were prevalent in PF. The two most prevalent CCs in the FPE and FFP compartments, CC9 and CC121, were rarely or never detected in PF. No CC was exclusively associated with the pork sector. Three CCs (CC5, CC6, and CC2) were considered ubiquitous, because they were observed in comparable proportions in all food production sectors. The two most prevalent CCs in all sectors were CC9 and CC121, but their distribution was disparate. CC9 was associated with meat products and food products combining several food categories, whereas CC121 was not associated with any given sector. Based on these results, CC121 is likely able to colonize a larger diversity of food products than CC9. Both CCs being associated with the food production suggests, that certain processing steps, such as slaughtering or stabilization treatments, favor their settlement and the recontamination of the food produced.

## Introduction

*Listeria monocytogenes* (*L. monocytogenes*) is one of the main causative agents for foodborne infections in Europe in terms of severity of the illness and fatality rate (EFSA-ECDC, 2015). In France, listeriosis causes less than 0.1% of foodborne illnesses, but has the highest rate of mortality (20–30%) and hospitalizations (98.9%) among foodborne infections (Goulet et al., 2013; Van Cauteren et al., 2017). A significant increase in listeriosis cases was recorded from 2011 to 2015 in France (Tourdjman et al., 2014; EFSA-ECDC, 2015). Among the large variety of food products that can be contaminated by *L. monocytogenes* (Henri et al., 2016), MP — and more specifically pork meat — are regularly reported as contaminated, with a prevalence of up to 12% in raw products (Roussel et al., 2010; Kerouanton et al., 2011). Several pork products are classified as at risk by the French public health authority (Tourdjman et al., 2014). Understanding the origin of these contaminations remains an important public health issue.

*Listeria monocytogenes* can be found in diverse ecological niches: natural and farm environments, animals, food and humans (Vivant et al., 2013) and is able to survive for long periods of time in unfavorable environments that do not allow the strains to grow (Carpentier and Cerf, 2011). These factors make its circulation difficult to trace. A better understanding of *L. monocytogenes* genetic population structure may help to characterize the circulation routes. Multilocus sequence typing (MLST) has been recognized as the key molecular method for investigating the population structure of *L. monocytogenes* strains (Chenal-Francisque et al., 2011; Cantinelli et al., 2013; Henri et al., 2016; Maury et al., 2016). It provides a portable and standardized nomenclature, based on the nucleotide sequence of seven housekeeping genes. It classifies the strains by sequence types (STs), defined as the unique association of alleles from the seven housekeeping genes, and by clonal complexes (CCs), defined as a cluster of STs sharing at least six alleles.

In France, the major CCs responsible for clinical cases are present in food samples (Maury et al., 2016). In particular, CC1, CC2, CC4, and CC6 are strongly associated with a clinical origin and the most likely to cause disease, in particular human central nervous system infections or maternal-neonatal listeriosis (Maury et al., 2016). Other CCs, such as CC9 and CC121, are more often isolated in highly immuno-compromised patients (Maury et al., 2016) and are preferentially associated with food production sectors (Henri et al., 2016; Maury et al., 2016). CC9 and CC121 are prevalent across all food production sectors (Henri et al., 2016). The introduction sources of these CCs in the food supply chain are not well understood. To date, few data are available on the distribution of these two food-associated CCs in other compartments upstream from the food supply chain, in particular in animals and farm environments.

The pork production sector has been hit hard by a series of *L. monocytogenes*-related food poisoning outbreaks in the past in France, from 1992 to 2015 (Jacquet et al., 1995; Goulet et al., 1998; de Valk et al., 2001; Tourdjman et al., 2014, 2016; Moura et al., 2017). An overview of *L. monocytogenes* genetic diversity, along the entire pig and pork production chain, is needed to improve food safety, identify the contamination routes and prevent human infections. Numerous studies have been successively carried out to investigate the genetic diversity of *L. monocytogenes* pig and pork strains in France. However, the strains were isolated from only one or two compartments of the pork production chain (Giovannacci et al., 1999; Chasseignaux et al., 2001; Thevenot et al., 2005, 2006; Hong et al., 2007).

Here, we focused on 687 *L. monocytogenes* strains isolated in France along the entire pig and pork production chain, from PF to FFP, and we analyzed the population genetic structure of these strains. All strains were typed by pulsed-field gel electrophoresis (PFGE), and then assigned to an MLST CC, using a mapping method specifically developed for this study. This method enabled to use the large amount of PFGE data collected for national surveillance to study the genetic population structure of *L. monocytogenes*. The distribution and prevalence of CCs in the different pig and pork production chain compartments were compared. Then, the CCs obtained were compared with those obtained from 1106 strains isolated from the other main food production sectors in France.

## Materials and Methods

### Complete Panel of 1793 Strains

**FIGURE 1:**
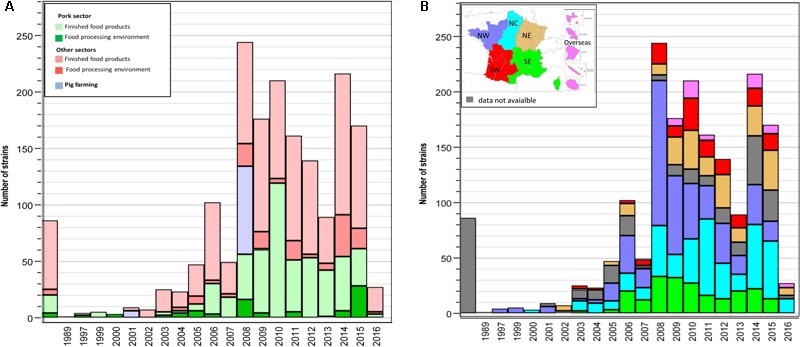
**(A)** Breakdown of the occurrence of *L monocytogenes* in the Pig farming (PF), Food-processing environment (FPE) and in finished food products (FFP) compartments per year. The green bars show isolates from FPE or FFP including pork meat (dark green, FPE; light green, FFP), the blue bars show the isolates from the PF compartment, the red bars show the isolates that do not include pork meat (dark red, FPE; light red, FFP). **(B)** Breakdown of the occurrence of *L monocytogenes* isolated from the PF, FPE, and FFP compartments per year according to their geographic origin throughout France. Five roughly equal regions were defined in terms of area and human population: Northwest (NW), Southwest (SW), North center (NC), Northeast (NE), Southeast (SE) and overseas. The map shows the defined regions.

The complete panel of investigated strains included 1793 strains isolated in France from the pig and pork production chain and the other food sectors. The year of isolation was known for 1707 strains. These strains were isolated over 27 consecutive years with 80% isolated between 2008 and 2015. From 2008 to 2015, the number of strains isolated every year from the pork sector ranged between 41 and 119. For the other food production sectors all together, the number of strains ranged between 47 and 162 per year (**Figure 1A**).

Two geographic levels were considered, the 101 *départements* that are administrative divisions rational in terms of surface and economic activity. For instance the analysis and centralization of *L. monocytogenes* strains is managed at *département* level. Based on *département* borders, five larger regions were defined as roughly equal in terms of surface area and human population (**Figure 1B**). The identity of the French *département* was known for 1512 strains (84.3%). The geographic distribution covered 93 of the 101 French *départements*. At region level, from 2008 to 2015, between 10 and 131 strains were isolated per year and per region (**Figure 1B**). Excluding overseas territories, the distribution of the isolates was similar among regions within the pork sector, with values ranging from 66 to 140 strains (Supplementary Table S1). The distribution of the isolates among regions in the other sectors was variable. A minimum of 4 strains was reported from the sector Fruit, vegetables, cereals, and herbs in the south east region and a maximum of 104 strains was reported from the sector Fish and fishery products in the north center region (Supplementary Table S1).

The panel did not include any epidemiologically duplicate strains. Duplicate strains were defined as strains sharing indistinguishable PFGE profiles, isolated the same year and provided by the same food business operator or the same diagnostic food laboratory. When the latter information was not available, the *département* was used instead. All the strains for which the year of isolation was not known had different PFGE profiles.

### Panel of 687 Strains Isolated From the Pig and Pork Production Chain

This panel was compiled as part of the ARMADA joint technological unit, a five-year collaborative French project between two partners: the French National Reference Laboratory (NRL) for *L. monocytogenes* (at the French Agency for Food, Environmental and Occupational Health & Safety, ANSES) and the French Institute for Pig and Pork Industry (IFIP). The IFIP strains were collected through research projects with corporate clients of IFIP. The ANSES strains were isolated during official sampling carried out by competent authorities (Roussel et al., 2010, 2012), national surveys (Roussel et al., 2014a), via research projects (Kerouanton et al., 2011) or collaboration with private laboratories or food business operators.

The 687 strains were isolated from three different compartments of the pig and pork production chain: PF, FPE, and FFP.

#### Strains From Pig Farming (PF)

A total of 85 PF strains were considered. Most strains (*n* = 78) were collected in 2008, from pig feces, during a survey carried on 73 farrow-to-finish pig farms located in Brittany, France (Boscher et al., 2012). Another part of the panel (*n* = 6) was sampled from pig skin swabbing on farms. One strain was isolated from pig manure. The four *départements* of the Brittany region accounted for nearly 58% of the French pig production in 2008 (IFIP, 2010).

#### Strains From the Food Processing Environment (FPE)

The 84 FPE strains were isolated from surface sampling carried out in 37 food factories, or butchery workshops that process pork meat, in 18 of the 101 French *départements*.

#### Strains From Finished Food Products (FFP)

A total of 518 FFP strains, isolated at the processing plant or at the point of sale, were considered. These strains were isolated in 86 of the 101 French *départements*.

In this compartment two groups were defined:

(i) unprocessed meat (UM), including, fresh meat, minced meat and meat preparations (*n* = 248);
(ii) meat products (MP) including, non-heat-treated or heat-treated products (*n* = 270).

### Panel of 1106 Strains Isolated From Other Food Production Sectors

For strains from other food production sectors, none were isolated from farms or primary production. In all, 963 strains (87.0%) were isolated from the FFP compartment and 143 strains (13.0%) from the FPE compartment. The strains came from five main food sectors: “MP” (excluding pork meat) (*n* = 284), “Milk products” (*n* = 287), “Fish and fishery products” (*n* = 237), “Food products combining several food categories” (*n* = 205) and “Fruit, vegetables, cereals, and herbs” (*n* = 67). Finally, 26 strains were isolated from FPE or FFP without assignment to a specific food sector.

### Molecular and Conventional Serotyping

Serotyping was done on 1691 strains including 523 typed by conventional serotyping (Seeliger and Höhne, 1979), and 1168 typed by molecular serotyping (Doumith et al., 2004). For 102 strains collected from research projects the serotype was not available. Here, conventional serotyping results are expressed using the molecular serotyping nomenclature, both nomenclatures being concordant (Kerouanton et al., 2010).

### Molecular Typing Database and PFGE Profile Interpretation

As part of the ARMADA project, IFIP and ANSES worked together to harmonize their PFGE typing methods. The PFGE and PFGE profile interpretation was performed according to Roussel et al. (2014b). A shared database (BioNumerics software, version 7.6 Applied Maths, Kortrijk, Belgium) was used to compare PFGE profiles and to centralize detailed epidemiological (sampling stage, context, sources, food sector, and food product) and genotypic (serotyping and PFGE) data (Felix et al., 2016, 2017).

All *Asc*I-*Apa*I PFGE profiles were analyzed according to the ANSES profile interpretation protocol (Roussel et al., 2014b) and interpreted together. A combined PFGE cluster was defined as a group of strains for which the PFGE profiles showed 85% similarity [average similarity of *Asc*I-*Apa*I profiles, unweighted pair group method with arithmetic average (UPGMA), similarity calculated using the Dice coefficient, tolerance, and optimization set to 1%]. Each cluster was numbered consecutively, starting from “1.” Thereafter, the numbers of the 26 most prevalent clusters were replaced by letters, starting with the letter “A” and assigned in decreasing order of prevalence.

On the basis of the PFGE 85% similarity clusters, the ability of PFGE to discriminate the strains between UM and MP groups was calculated using the Simpson diversity index (ID) (Hunter and Gaston, 1988), with BioNumerics.

### Mapping MLST/PFGE Clusters

We created a mapping protocol to identify matches between CCs and PFGE clusters. To do so, we used the typing data from 396 strains, previously typed in our laboratory using both PFGE and MLST (Henri et al., 2016) methods. These typing data were associated here along with those from the complete strain panel (1793 strains) to deduce the CCs from their PFGE clusters.

We used the adjusted Wallace (AW) coefficient (Severiano et al., 2011) to measure the directional concordance from PFGE to MLST. In Henri et al. (2016), this coefficient was calculated on the 396 strains, based on the STs and PFGE 80% similarity clusters. The AW obtained was 0.853, 95%, CI_95%_ [0.874–0.920]. Here, this coefficient was recalculated on the same panel of 396 strains, based on the CCs and PFGE 85% similarity clusters. The AW obtained was 0.92, CI_95%_ [0.798–0.907]. Based on this result, we decided to set our mapping on CCs and PFGE 85% similarity clusters.

The strains sharing the same PFGE cluster were considered as belonging to the same CC when they clustered with at least 85% similarity with a strain previously typed by MLST. When two clusters were associated with the same CC, the clusters were merged into one cluster. When several strains from different CCs were part of the same cluster, the CCs were merged (e.g., the cluster G encompassed two CCs and was named CC4-CC217). When the strains did not share PFGE clusters with a strain typed by MLST, the CC was not assigned.

### Statistical Analysis

The distribution of the mapped CCs within the compartments and the food sectors was described using counts and percentages. To determine if the proportions of a CC among compartments/food sectors were equivalent, the “bayes.prop.test” function of the Bayesian First Aid R package (Kruschke, 2011; Bååth, 2014) was used. A *p*-value above 0.025 was considered as significant. The statistical tests were performed using the R software version 3.2.

### Distribution Analysis

The distribution of the CCs was compared, first between the three compartments of the pig and pork production chain, and second between the pork sector and the other food production sectors. The “pork” sector only included the FPE and FFP strains. PF strains were not included in the comparison because the other food sectors did not include strains from primary production. CCs associated with one specific compartment/sector were highlighted as well as CCs homogenously distributed throughout the various compartments/sectors.

## Results

### Strain Serotype Diversity

For pig and pork production, the major serotype was IIa in all three compartments. IIc strains were rare in the PF compartment, and were predominantly isolated in the FPE and FFP compartments (**Table 1**).

**Table 1:** Distribution of the 1793 strains isolated from food production sectors according to their molecular serotype.

			Number (%) of strains according to the food sector							
Serotype		Pig farming (%)	Pork sector (excluding pig farming strains)	Meat products sector (excluding pork meat)	Milk products sector	Fish and fishery products sector	Food products combining several food categories sector	Fruit, vegetables, cereals, and herbs sector	Without assignment to a specific food sector	Total number (%)
Lineage II	IIa	35 (41.2)	312 (51.8)	139 (48.9)	122 (42.5)	159 (67.1)	105 (51.2)	43 (64.2)	16 (61.5)	931 (51.9)
	IIc	1 (1.2)	134 (22.3)	63 (22.2)	7 (2.4)	8 (3.4)	31 (15.1)	1 (1.5)	3 (11.5)	248 (13.8)
	N/A	–	–	6 (2.1)	29 (10.1)	18 (7.6)	11 (5.4)	2 (3)	–	66 (3.7)
Lineage I	IIb	31 (36.5)	67 (11.1)	23 (8.1)	31 (10.1)	19 (8)	20 (9.8)	5 (7.5)	2 (7.7)	198 (11)
	IVb	18 (21.2)	89 (14.8)	50 (17.6)	75 (26.1)	30 (12.7)	33 (16.1)	15 (22.4)	4 (15.4)	314 (17.5)
	N/A	–	–	3 (1.1)	23 (8)	3 (1.3)	5 (2.4)	1 (1.5)	1 (3.8)	36 (2)
Total		85 (100)	602 (100)	284 (100)	287 (100)	237 (100)	205 (100)	67 (100)	26 (100)	1793 (100)

For the pork sector and all the other food sectors, IIa was the major serotype. The proportion of IIc strains was equivalent between the pork sector and the MP sectors 22.3 and 22.2% of the strains, respectively, had this serotype (**Table 1**).

### Strain Genetic Diversity From the Complete Panel (1793 Strains)

**FIGURE 2:**
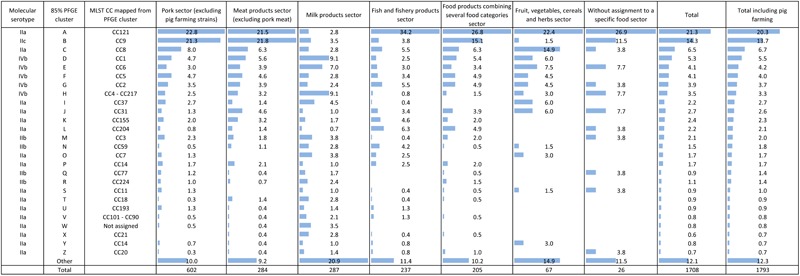
Distribution of the 26 major mapped clonal complexes (CCs) in the pork production sector and in other food production sectors.

The 1793 strains were distributed into 111 PFGE 85% similarity clusters, including 26 major clusters. Nine major clusters accounted for the majority of the strains (67% of the complete panel). The two most prevalent clusters A and B accounted for 20.3 and 13.7% of the strains, respectively (**Figure 2**). They corresponded to the MLST-mapped CCs, CC121 and CC9, respectively (**Figure 2**). The seven following clusters C, D, E, F, G, H, and I corresponded to the mapped CCs CC8, CC1, CC6, CC5, CC2, CC4-CC217, and CC37, respectively (**Figure 2**).

The majority of CC9 strains (*n* = 245) were of serotype IIc (*n* = 231). The majority of the IIc strains (*n* = 248) were CC9 (*n* = 231).

### Strain Genetic Diversity From Pig Farming and Pork Production (687 Strains)

#### Comparison of the MLST Clonal Complex Distributions Between the Three Compartments

**FIGURE 3:**
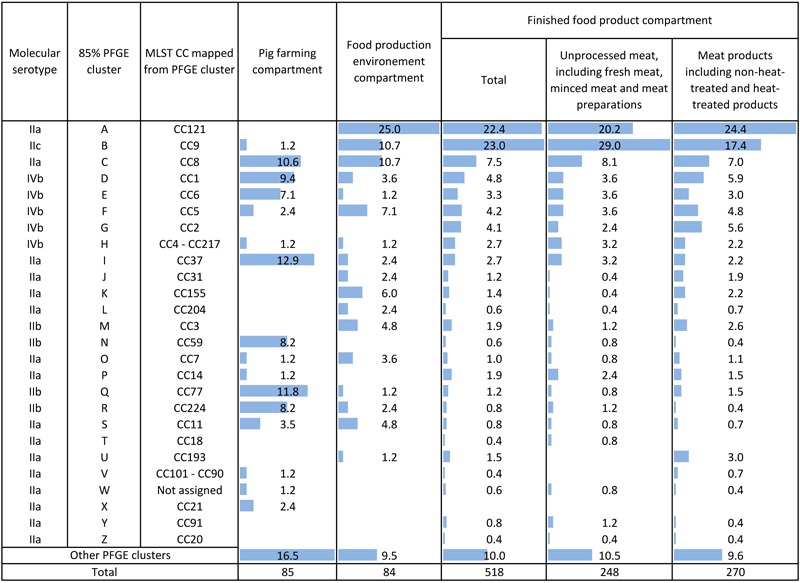
Distribution of the 26 major mapped clonal complexes (CCs) within the pig and pork meat production chain.

CC121 was not observed among the strains identified in the PF compartment, but was one of the most prevalent CCs in the FPE (25%) and FFP (22.4%) compartments (**Figure 3**). The distribution of CC121 was comparable in these two compartments (*p*-value > 0.27). CC9 was associated with the FFP, but not with PF or FPE compartments (*p*-value < 0.001). CC37, CC77, and CC59 were associated with the PF, but not with the FPE or FFP compartments (*p*-value < 0.004). The distributions of CC8, CC1, CC5 (*n* > 30) and CC6, CC4-CC217, CC7 (*n* < 30) were comparable in the three compartments (*p*-value > 0.038) (**Figure 3**).

#### Pig Farming

CC37, CC77, CC8, CC1, CC224, CC59, and CC6 were the seven most prevalent CCs, together accounting for 68.2% of the strains. CC37, CC77, CC8 accounted for 12.9, 11.8, and 10.6% of the strains analyzed, respectively (**Figure 3**).

#### Food Processing Environment

CC121, CC8, CC9, CC5, CC155, CC3, and CC11 were the most prevalent strains, together accounting for 69% of the strains. CC121, CC8, CC9 accounted for 25, 10.7, and 10.7% of the strains analyzed, respectively (**Figure 3**).

#### Finished Food Products

CC9, CC121, CC8, CC1, CC6, CC5, CC2, CC4-CC217, and CC37 were the most prevalent strains, together accounting for 74.7% of the strains analyzed. CC9, CC121, and CC8 accounted for 23, 22.4, and 7.5% of the strains analyzed, respectively.

The UM and MP groups included, respectively, 38 and 43 PFGE clusters. The Simpson’s index of diversity obtained for the MP group was significantly higher than that for UM, with values of 0.894, CI_95%_ [0.884–0.904] and 0.862, CI_95%_ [0.849–0.875], respectively.

The most prevalent CCs in the UM and MP groups were CC9 (29.0 and 17.4%), CC121 (20.4 and 24.4%), and CC8 (8.1% and 7.0%). The prevalence of CC9 significantly decreased between the UM and MP groups (*p*-value < 0.001).

### Strain Genetic Diversity Compared Between the Pork Sector and the Other Food Production Sectors

No CC was exclusively associated with the Pork sector. The distributions of CC5, CC6, and CC2 were comparable between the Pork sector and the five other food production sectors (*p*-value > 0.038). The other CCs (except cluster W) were found in the same proportion in the pork sector and at least two other food production sectors (**Figure 2**, *p*-value > 0.028). CC121, CC9, CC8, CC1, CC4-CC217, and CC59 were found in all food production sectors.

The distribution of the CC121 was comparable in the Pork sector and the MP, Food products combining several food categories and Fruit, vegetables, cereals, and herbs sectors (*p*-value > 0.118). Compared with the Pork sector, the prevalence of CC121 was 10 times lower in the Milk products sector (*p*-value < 0.001), but one-third higher in the Fish and fishery products sector (*p*-value < 0.001) (**Figure 2**).

The distribution of CC9 was comparable between the Pork sector and the following two sectors: MP and Food product combining several food categories (*p*-value > 0.028). In contrast, CC9 was rarely found in the three other food sectors (*p*-value < 0.001) (**Figure 2**). Among the 31 CC9 strains isolated from the Food products combining several food categories sector, only seven included meat. Nine were isolated from the FPE and supplied by two different private companies (*n* = 9). Nine others were isolated from readymade meals or salads including fish or cheese (*n* = 9). The remaining strains (*n* = 6) were isolated from combined food products for which the composition was not specified.

The prevalence of CC1, CC6, and CC4-CC217 was comparable in the Pork sector and in the four other production sectors (*p*-value > 0.026), except the Milk products sector in which these CCs were more abundant (*p*-value < 0.006) (**Figure 2**).

## Discussion

First, this study aimed to understand the genetic diversity of *L. monocytogenes* strains isolated along the pig and pork production chain in France and to compare it between the three compartments: PF, FPE, and FFP. The 687 strains were isolated over 20 consecutive years from retail stores and hundreds of different pork processing plants. To our knowledge, this study represents the largest and the most representative study ever performed in France on this production chain. Former studies investigated only one or two compartments of the pig and pork production chain: slaughterhouses, meat processing plants (Giovannacci et al., 1999; Chasseignaux et al., 2001; Thevenot et al., 2005, 2006), and retail stores (Hong et al., 2007). The highest number of consecutive years for sampling was 2 years (Hong et al., 2007). The highest numbers of strains analyzed and sample plants were 1028 and 13, respectively (Thevenot et al., 2005, 2006). Moreover, in contrast to our work, previous authors included strains considered as duplicates according to our criteria (strains sharing indistinguishable PFGE profiles, isolated the same year and provided by the same food business operator or the same diagnostic food laboratory). If we remove the duplicates from the Thevenot et al. (2006) study, 90% of the strains would not be included.

One of the main results obtained here is that the major CCs of pork strains were not equally distributed among the three compartments. Three CC (CC37, CC59, and CC77) strains were rarely found in the FPE and FFP compartments, but were prevalent and associated with the PF compartment. CC37, the most prevalent CC in the PF compartment in our study, was frequently isolated from Austrian soil and river environments (Linke et al., 2014), from ruminant farm environment (Dreyer et al., 2016) and from farm/wild environments or animals (Haase et al., 2014). CC37 is likely better adapted to pig farms than to the pork production environment.

The two most prevalent CCs in the FPE and FFP compartments, CC9 and CC121, were not — or only rarely — found in the PF compartment. This result echoed the outcomes of Haase et al. (2014) study reporting 3.5% of CC9 and 1% of CC121 in a panel of 312 strains from farm/wild environments or animals. No CC9 and no CC121 were reported from the study of Dreyer et al. (2016), which considered 248 strains isolated from ruminant farms over 2014–2015 period and from the study of Linke et al. (2014), which considered 27 strains isolated from Austrian soil over the 2007–2009 period.

A salient fact we observed in the present study is the near-perfect overlap in the CC9 and IIc strains, as already demonstrated by Ragon et al. (2008), Haase et al. (2014), Martin et al. (2014), or Henri et al. (2016). In the FPE and FFP compartments, there was a high prevalence of CC9 and by correlation of IIc serotype strains (22.3%). It corroborated the results from other studies conducted on the pork production chain in France (Giovannacci et al., 1999; Thevenot et al., 2005, 2006; Hong et al., 2007), in Spain (Ortiz et al., 2010), and also in Italy (Prencipe et al., 2012; Meloni et al., 2014; Morganti et al., 2015). In these studies, the proportion of IIc strains was on average 28.5%, with the highest proportion being 50% (Prencipe et al., 2012). All these data confirm that the pork production chain is often contaminated by IIc strains.

In the FFP compartment, strain genetic diversity was lower in the UM than in the MP group. This difference in diversity may be due to: (i) the diversity of *L. monocytogenes* strains contaminating raw products and ingredients; (ii) the abundance of niches, due to difficult-to-clean surfaces, in the production line and (iii) the ability of some strains to survive cleaning and disinfection procedures and to remain in the factory environment (Chasseignaux et al., 2001; Thevenot et al., 2006; Carpentier and Cerf, 2011).

Second, this study aimed to compare the genetic diversity between the Pork sector and the other food production sectors on a strain panel collected over 27 years of sampling, from hundreds of processing facilities and retail stores. Among the major CCs obtained, we distinguished three CCs (CC5, CC6, and CC2) considered ubiquitous, because they were found in comparable proportions in all sectors, from seven other CCs found in all the food sectors, but for which distribution was disparate (CC121, CC9, CC8, CC1, CC4-CC217, and CC59).

For instance, CC9 was rarely found in the three following sectors: Fish and fishery products, Milk products, and Fruits, vegetable, and herbs. CC9 was mainly associated with three sectors: Pork, MP, and Food products combining several food categories. In this latter sector, some CC9 strains were isolated from (i) a large variety of food products with or without meat; (ii) from FPE in factories producing readymade meals. This result suggests that the FPE may be responsible for cross contaminations between food products that include and do not include meat.

In a three-year multi-food sector study conducted in Ireland (Leong et al., 2017), a pulsotype, named P32, was observed primarily in meat and, to a much lesser degree, in dairy products. The P32 PFGE profile provided by the Irish study’s authors mapped to CC9 in the present study. CC9 was also predominantly isolated from MP in Spain (Martin et al., 2014), Switzerland (Ebner et al., 2015), and in Europe (Nielsen et al., 2017). CC9 contamination was shown for mammalian meat production, regardless of meat type (Martin et al., 2014; De Cesare et al., 2017). From all these results, it appears that CC9 finds favorable settlement conditions in the meat production sector. In the present study, CC9 was rarely encountered in the PF compartment suggesting that the contamination is likely not related to the primary contamination of livestock animals. This result needs confirmation on a larger strain panel.

As descried previously that almost all IIc strains are part of the CC9. Several studies report increased detection of IIc strains at the slaughterhouse, after carcass dressing and prior to transfer to the ultraclean meat processing area (Fravalo et al., 2013; Lariviere-Gauthier et al., 2014; Neira et al., 2015). These results may indicate that CC9 has found a favorable ecological niche at certain steps in the slaughtering process. These results echoed the studies of Thevenot et al. (2005, 2006), in which the number of 1/2c serotype strains decreased after curing or salting pork meat, whereas the number of 1/2a strains increased. Once meat is contaminated at the slaughterhouse, CC9 does not seem to find favorable conditions for growth in the subsequent steps of meat processing.

In contrast to CC9, CC121 was not associated with a given food sector. However, CC121 was the most prevalent in the Fish and fishery products, Pork, and MP sectors. These results echoed studies conducted in the smoked fish production sector in Denmark where CC121 is prevalent (Wulff et al., 2006; Holch et al., 2013). Two other studies conducted nationally on the food production chain, including milk and meat production (Ebner et al., 2015; Leong et al., 2017), reported CC121 strains mostly from MP or meat processing environments. A study including fish, meat and milk ready-to-eat products across Europe (Nielsen et al., 2017) reported a high prevalence of CC121 in fish and meat ready-to-eat products. These data suggest that CC121 strains may be more specifically adapted to a given production sector or processing steps common to the fish and meat production sectors. We therefore suggest the following scenario. CC121 strains may preferentially contaminate food products characterized by reduced total bacterial flora. For instance, meat and fish production is characterized by low total bacterial flora in the raw materials (absence of bacteria in flesh). Furthermore, a series of stabilization treatments are applied during the transformation process of fish and MP to avoid contamination. These factors likely promote the settlement of CC121 in the food production chain where chemical stresses are used for bacterial load reduction or disinfection. Recent investigations have suggested that two genetic factors in CC121 strain genomes, the stress survival islet 2 (Harter et al., 2017) and transposon Tn6188 (Muller et al., 2013), provide tolerance to alkaline and oxidative stresses and to benzalkonium chloride, respectively.

The mapping method developed here (correspondence between CCs and PFGE clusters) has been used in the database set up in the ARMADA joint technological unit (Felix et al., 2016, 2017). As performed in this study, mapping a large amount of historical PFGE data with CCs will help to (i) obtain a wide view of strain genetic diversity circulating in different food sectors; (ii) assess the risk represented by *L. monocytogenes*, according to the degree of virulence of the CC and the exposure of French consumers.

## Conclusion

The results obtained in this study led to a better understanding of the structure of the *L. monocytogenes* population isolated from the pig and pork production sector and will contribute to the improvement of the management of the health risks associated with *L. monocytogenes* in this major food sector. CC9 and CC121 are associated with food production, most likely because processing steps, such as slaughtering or stabilization treatments, favor their settlement and recontamination of the food produced. Both results indicate that processing steps are likely the source point of contamination. Our results corroborate studies that recommend targeted deep-cleaning procedures to reduce contamination in processing plants (Hammons et al., 2017).

## Author Contributions

SR and BF planned the project. BF was in charge of the PFGE data analysis, interpretation, and the design, and the performance of the whole study. CF was in charge of the acquisition and analysis of the food processing environment data and the references and framework definition for pig and pork compartments. AM was in charge of PFGE production and interpretation. LG was in charge of supervision of statistical analysis. EB was in charge of analysis of pig farming data. AK was in charge of the analysis of unprocessed pork meat data. BF, CF, and SR drafted the manuscript and all authors read, commented, and approved the final manuscript.

## Conflict of Interest Statement

The authors declare that the research was conducted in the absence of any commercial or financial relationships that could be construed as a potential conflict of interest.

## Abbreviations

FFP: finished food products
FPE: food processing environment
MP: meat products
PF: pig farming
UM: unprocessed meat
